# The Application of Graphene Field-Effect Transistor Biosensors in COVID-19 Detection Technology: A Review

**DOI:** 10.3390/s23218764

**Published:** 2023-10-27

**Authors:** Qin-Hong Liang, Ban-Peng Cao, Qiang Xiao, Dacheng Wei

**Affiliations:** 1 Jiangxi Key Laboratory of Organic Chemistry, Jiangxi Science and Technology Normal University, Nanchang 330013, China; lllqh123@126.com (Q.-H.L.); xiaoqiang@tsinghua.org.cn (Q.X.); 2 State Key Laboratory of Molecular Engineering of Polymers, Department of Macromolecular Science, Fudan University, Shanghai 200433, China

**Keywords:** GFET biosensor, COVID-19, biological sensors, biological detection

## Abstract

Coronavirus disease 2019 (COVID-19) is a disease caused by the infectious agent of severe acute respiratory syndrome coronavirus type 2 (SARS-CoV-2). The primary method of diagnosing SARS-CoV-2 is nucleic acid detection, but this method requires specialized equipment and is time consuming. Therefore, a sensitive, simple, rapid, and low-cost diagnostic test is needed. Graphene field-effect transistor (GFET) biosensors have become the most promising diagnostic technology for detecting SARS-CoV-2 due to their advantages of high sensitivity, fast-detection speed, label-free operation, and low detection limit. This review mainly focus on three types of GFET biosensors to detect SARS-CoV-2. GFET biosensors can quickly identify SARS-CoV-2 within ultra-low detection limits. Finally, we will outline the pros and cons of the diagnostic approaches as well as future directions.

## 1. Introduction

In 2019, a novel coronavirus as a kind of severe acute respiratory syndrome coronavirus (SARS-CoV-2) swept the world, causing a respiratory disease which is named coronavirus disease 2019 (COVID-19) [1,2]. COVID-19 is reportedly transmitted by airborne and contact-transmission respiratory droplets [3,4]. The methods for diagnosing COVID-19 can be roughly divided into three categories [5]: chest CT scanning [6], serological testing [7,8], and nucleic acid testing [9]. Chest CT scanning is limited to recognizing the virus type and is only available in hospitals. Serological testing is not suitable for early diagnosis of infection because the number of antibodies in our bodies gradually increases for at least a week after being infected with COVID-19 [10,11,12]. The nucleic acid detection method requires skilled professionals, and it takes 4–6 h to obtain the results [13]. Real-time reverse transcription-polymerase chain reaction (RT-PCR) remains the gold standard for COVID-19 diagnosis. However, RT-PCR requires a gene amplification process, tedious preparation steps, expensive equipment, specialized laboratories, and technicians, which reduces the efficiency of the test [14]. Therefore, developing a real-time, fast, accurate, easy-to-operate, and low-cost diagnostic technology is one of the key challenges in the fight against COVID-19.

**Figure 1:**
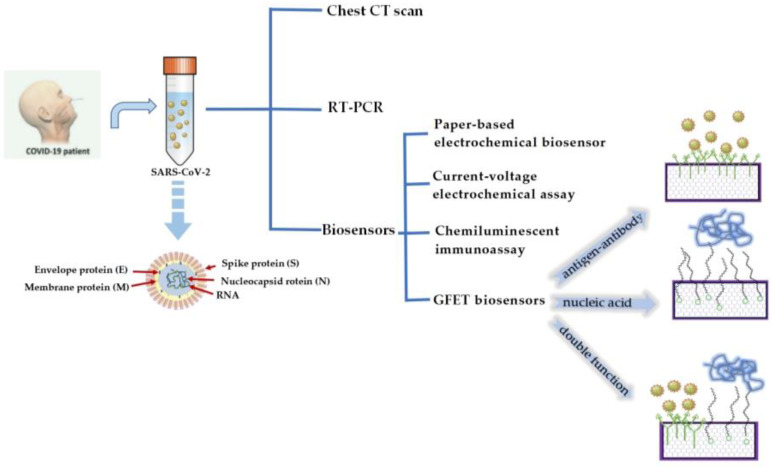
Several detection methods for COVID-19 and the schematic diagram of a GFET biosensor for detecting SARS-CoV-2.

Biosensors with simple operation and rapid detection have been widely considered a superior alternative detection technology [15]. In recent years, an endless stream of biosensors has been studied [16]. There are many types of biosensors, such as field-effect transistor (FET) biosensors [17], optical biosensors [18], plasmon resonance biosensors [19], and electrochemical biosensors [20]. These biosensors have become a powerful new means of detecting various biomolecules for diagnostics. In particular, GFET biosensors have the advantages of high sensitivity, fast detection speeds, no labels, and low detection limits and have become the most promising technology for COVID-19 detection [21,22]. GFET biosensors detect SARS-CoV-2 through specific targets on the surface of the graphene channel. SARS-CoV-2 is a segmented, enveloped coronavirus family with a single-stranded RNA structure [23]. The genome inside the particle encodes four structural proteins. Viral RNA is coated with nucleocapsid (N) protein. In addition to nucleocapsid (N) proteins, there are spike proteins (S), membrane proteins (M), and envelope proteins (E), which are embedded in the lipid bilayer [24]. Therefore, SARS-CoV-2 can be detected by using nucleic acid molecules or structural proteins as targets for the specific reaction (Figure 1). Currently, GFET biosensors for detecting COVID-19 mainly use nucleic acid hybridization or antigen–antibody-specific reaction for detection.

Two-dimensional graphene has excellent electronic, optical, and mechanical properties, and it is an incredibly vast and diverse material for optoelectronics. It is used in diverse applications. Rehman’s group [25] developed directly grown graphene–silicon Schottky barrier solar cells using the co-doping technique. The nonvolatile nature of polymeric perfluorinated sulfonic acid macromolecules and their strong binding with HNO_3_ on graphene provides a solid platform for hole injection along with excellent stability for optoelectronic devices; Khan’s group [26] fabricated a GFET through photochemical reactions which demonstrated bipolar photoresponse. The N-doping of graphene through this efficient photochemical method can enhance its electrical and photoelectrical properties; Yao’s group [27] prepared graphene/graphitic carbon nitride heterojunctions for ultrasensitive terahertz biosensors and achieved ultrasensitive, multi-dimensional sensing of casein molecules. Graphene has another very important property: it usually strongly adsorbs biomolecules due to the p-stacking interactions between its hexagonal cells and the carbon-based ring structures widely present in bio/nano-molecules [28]. Therefore, GFET biosensors have ultra-sensitive performance in terms of sensing, which provides an ideal biosensing platform for disease detection.

## 2. GFET Biosensors

Graphene is a nanosheet with excellent performance; therefore, GFET biosensors can detect COVID-19 at ultra-low detection limits and in ultra-short time. With the rapid development of nanomaterials, they have proven to be suitable for biosensors. Graphene, gold, quantum dots, nanotubes, nanorods, and nanoparticles have become important carriers for the biosensor immobilization of biomolecules [29,30,31]. In recent years, graphene has become the best potential nanosheet for biosensors because of its excellent properties [32,33].

**Figure 2:**
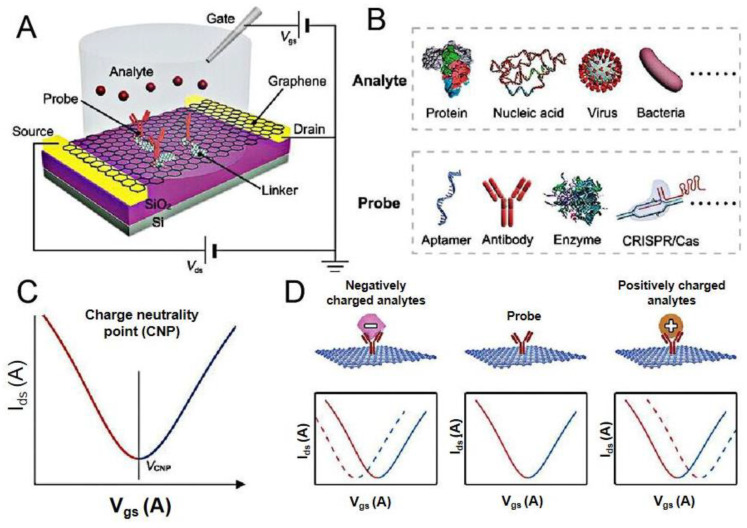
Working principles of GFET biosensors. (**A**) Schematic illustration of a liquid-gate GFET sensor. (**B**) The analytes include proteins, nucleic acids, viruses, and bacteria. The probes include aptamers, antibodies, enzymes, CRISPR/Cas. (**C**) Typical ambipolar transfer characteristics of graphene. (**D**) Sensing principle on the graphene surface: the binding of negatively (positively) charged analytes induced negative (positive) shifts in VCNP. Reprinted from [39], with permission from John Wiley and Sons.

Graphene, a kind of 2D carbon atomic flake material with a six-circular honeycomb lattice structure, has emerged as a widely studied 2D biosensor material. The main reason is its unique properties, such as its excellent electrical conductivity, thermal conductivity, high electron mobility, super-large specific surface area, electrochemical inertness, and biocompatibility [34,35,36]. GFET sensors have three main structures: a bottom/top gate, a source, and a drain (Figure 2). Biological molecules can be detected by modifying biometric elements on the graphene channel surface.

GFET biosensors generally have two fabrication methods. The first method is to fix biological receptors onto the surface of graphene. Due to the carbon atoms being exposed on the graphene surface, biological receptors can be fixed onto the graphene channel surface as detection targets through mutual forces. After specific binding, charge transfer changes the conductivity, converting biochemical changes into measurable electrical signals. However, the electrochemical inertness of graphene makes it less sensitive to biological receptors. Therefore, surface modification or functionalization is usually carried out through fixed nanoparticles, electrostatic adsorption, surface plasma pretreatment, and *π-π* interaction [37]. A common functionalization method is to immobilize specific targeted biological receptors on graphene surface through *π-π* interaction [22]. The second way is to fix the biological receptor into the fabricated cavity of the gate dielectric layer. The presence of biological molecules with specific dielectric constants inside this cavity changes the dielectric gate capacitance and causes a shift in the threshold voltage. A change in current due to threshold-voltage shift indicates the presence of the moiety [38,39].

GFET biosensors have become a powerful diagnostic method for the real-time and on-site detection of COVID-19 because of their advantages of high sensitivity, high selectivity, fast analysis speed, label free, low cost, miniaturization, and integration. Therefore, preparing high-quality graphene is an essential prerequisite for researching GFET biosensors. The typical preparation methods for graphene include the mechanical stripping method [40,41], the redox method [42,43], and chemical vapor deposition (CVD) [44,45]. The most commonly used methods are the redox method and CVD [43]. The excellent electrical, optical, chemical, and mechanical properties of graphene have attracted widespread attention from scholars. Many scholars look forward to finding simple, fast, and widely selected raw materials, controllable graphene patterning, and an environmentally friendly synthesis method. Tour’s group [46] first successfully prepared laser-induced graphene, which has the advantages of simple preparation, high efficiency, environmental protection, low cost, a wealth of raw materials, ease of functionalization, and surface modification. Therefore, laser-induced graphene is the latest technology discovered in recent years to prepare graphene [42].

Since the outbreak of the novel coronavirus, diagnosis of the disease has been crucial. Accurate and rapid detection methods can greatly prevent the spread of the epidemic in a short time. During this period, researchers developed various methods to detect it. Table 1 lists several detection methods for SARS-CoV-2 and shows the excellent performance of GFET biosensors in diagnosing SARS-CoV-2.

## 3. Application of GFET Biosensors in the Diagnosis of COVID-19

GFET biosensors have great potential for diagnosing and controlling disease transmission in COVID-19 and other biomolecular detections. Compared with traditional detection technology, GFET biosensors have certain advantages in the bedside maintenance of biomolecules because of their excellent performance.

### 3.1. GFET Biosensors Detect SARS-CoV-2 Based on Specific Antigen–Antibody Binding

**Figure 3:**
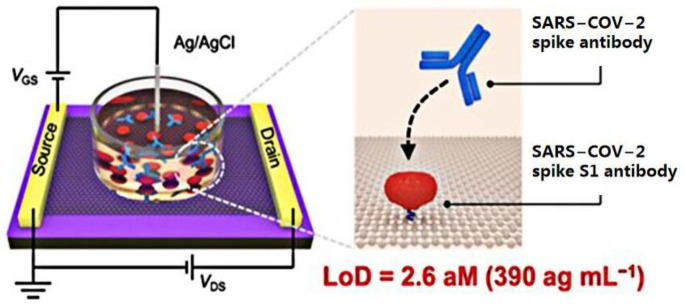
Schematic diagram of the GFET biosensor for detecting SARS-CoV-2 spike antibodies. Reprinted from [51], with permission from the American Chemical Society.

The coronavirus S protein is a large, multifunctional transmembrane fusion glycoprotein of the class I virus. The S protein is attached to the surface of the viral particle and determines the shape of the virus’ crown-like appearance. The coronavirus N protein promotes the assembly of viral particle and plays a role in the formation of the viral genome. After being infected with SARS-CoV-2, B lymphocytes or B cells produce five types of antibodies, IgA, IgG, IgM, IgD, and IgE, known as immunoglobulins [10]. Therefore, many researchers have detected SARS-CoV-2 by targeting antibodies. An ultra-sensitive GFET biosensor was prepared by Wei’s group [51]. The SARS-CoV-2 spike S1 protein was modified on the surface of the sensor to detect the spike S1 antibody (Figure 3). The SARS-CoV-2 spike S1 protein was immobilized on the surface of the graphene channel to realize biological functionalization. The strong specific binding between antibodies and proteins affected the concentration of the graphene channel medium and obtained a measurable electrical response. In this study, the GFET biosensor detection limit for the SARS-CoV-2 spike S1 antibody was as low as 2.6 × 10^−18^ M, and it took only 2 min to produce a diagnostic result.

**Figure 4:**
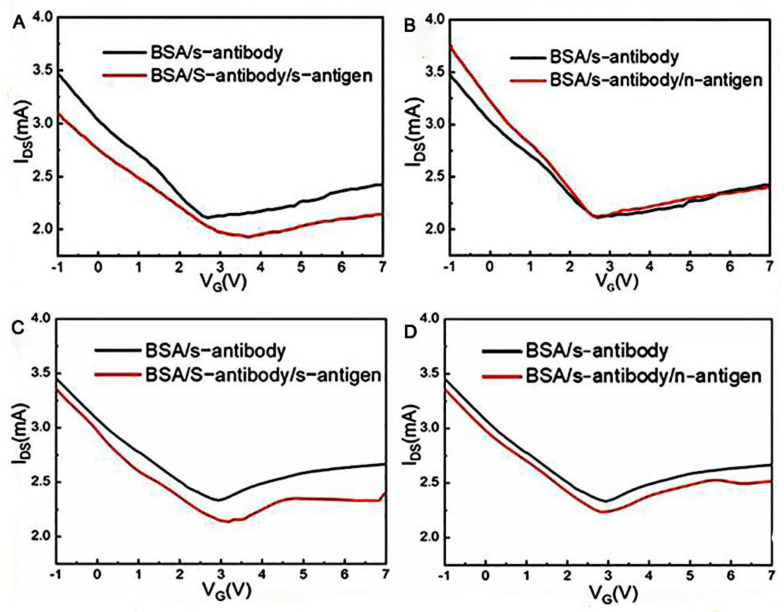
The virus detection performance of the laser-induced GFET. (**A**) Transfer characteristics of the laser-induced GFET biosensor responding to the complementary 1 pg/mL spike protein in PBS solution and (**B**) responding to the noncomplementary 1 pg/mL nucleocapsid protein in PBS solution. (**C**) Transfer characteristics of the laser-induced GFET biosensor responding to 1 pg/mL of complementary spike protein in human serum and (**D**) responding to 1 pg/mL noncomplementary nucleocapsid protein in human serum.

In recent years, laser-induced graphene technology has become a new way of preparing GFET biosensors. Cui’s group [52] first used lasers to manufacture a graphene channel, using a 450 nm UV laser of 840 MW, and the electrode region was manufactured using a UV laser of 900 MW. The graphene channel region obtained after laser radiation showed a spongy porous shape, which expanded its binding area with biomolecules. This research group has developed a one-step, simple, sensitive, and suitable method for the large-scale preparation of a laser-induced GFET biosensor. The SARS-CoV-2 spike antibody immobilized in graphene channels achieved rapid detection of the SARS-CoV-2 spike protein in 15 min at a detection limit of 1 pg/mL in phosphate-buffered saline (PBS) and 1 ng/mL in human serum, with high specificity for the target virus (Figure 4).

With the fusion of nanoparticles, GFET biosensors improve the detection performance for SARS-CoV-2. Novodchuk’s group [13] reported on a boron and nitrogen co-doped graphene oxide gel (BN-GO gel) sensor. The SARS-CoV-2 nucleocapsid protein antibody was immobilized on the surface of the BN-GO gel sensor, which could rapidly respond to the SARS-CoV-2 nucleocapsid protein within 4 min. The detection limit was up to 10 ag/mL.

**Figure 5:**
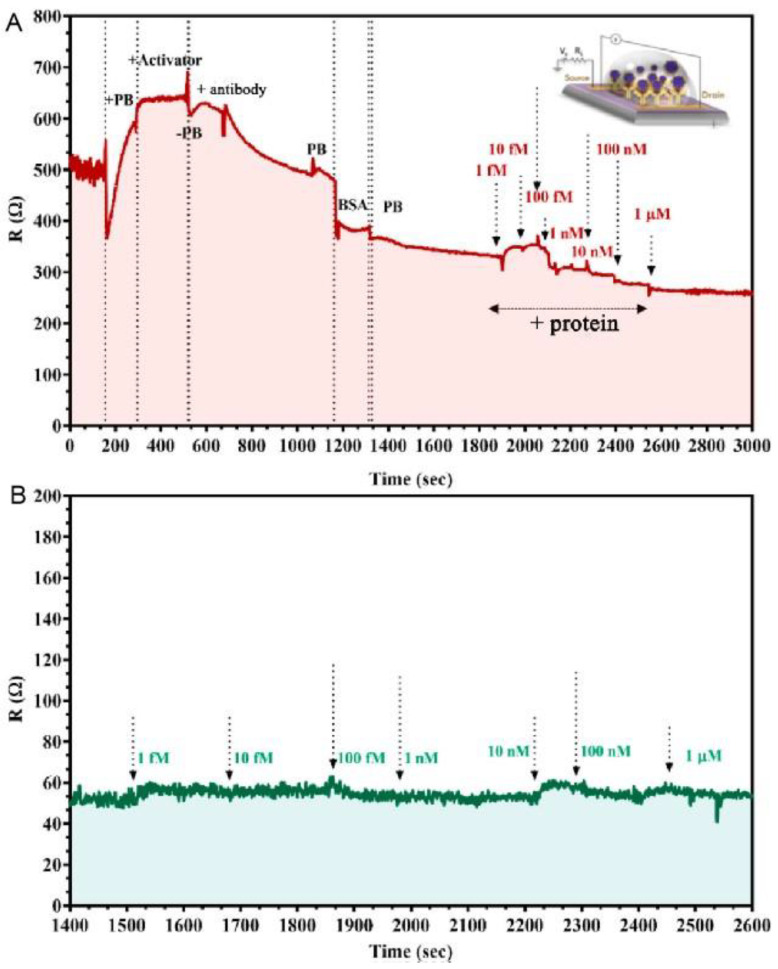
The kinetic response of the GFET device functionalized with the SARS-CoV-2 spike antibody at various concentrations of (**A**) SARS-CoV-2 spike protein added, ranging from 1 fM to 1 μM in 50 mM phosphate buffer (PB) (pH 7.2) and (**B**) MERS-CoV protein of various concentrations added (1 fM to 1 μM) in PB. Reprinted from [21], with permission from the American Chemical Society.

Shahdeo’s group [21] prepared GFET sensors on SiO_2_/Si substrates using transparent tape. The internally generated SARS-CoV-2 spike S1 antibody was immobilized on the carboxylic acid-activated graphene surface. The change in resistance generated by antibody–antigen interaction was monitored in response to the assay results (Figure 5). The results indicated that the device has high sensitivity and specificity and detects SARS-CoV-2 spike S1 proteins under conditions with a detection limit as low as 10 fM.

### 3.2. GFET Biosensors Based on Nucleic Acid Hybridization Detection of SARS-CoV-2

Nucleic acid detection is the most sensitive detection method for early viral infection and plays a key role in diagnosing and treating disease [53]. The RT-PCR method is the gold standard for detecting SARS-CoV-2, but the diagnostic process is complicated and time consuming. Therefore, many scholars tend to develop methods based on nucleic acid as a probe for detecting SARS-CoV-2. How to improve the sensitivity of the probe is an important problem. A lot of work has been carried out, mainly focused on the design of the probe and the development of sensing materials and new sensing mechanisms. In bioassays, different configurations are designed to improve the binding affinity with the target.

**Figure 6:**
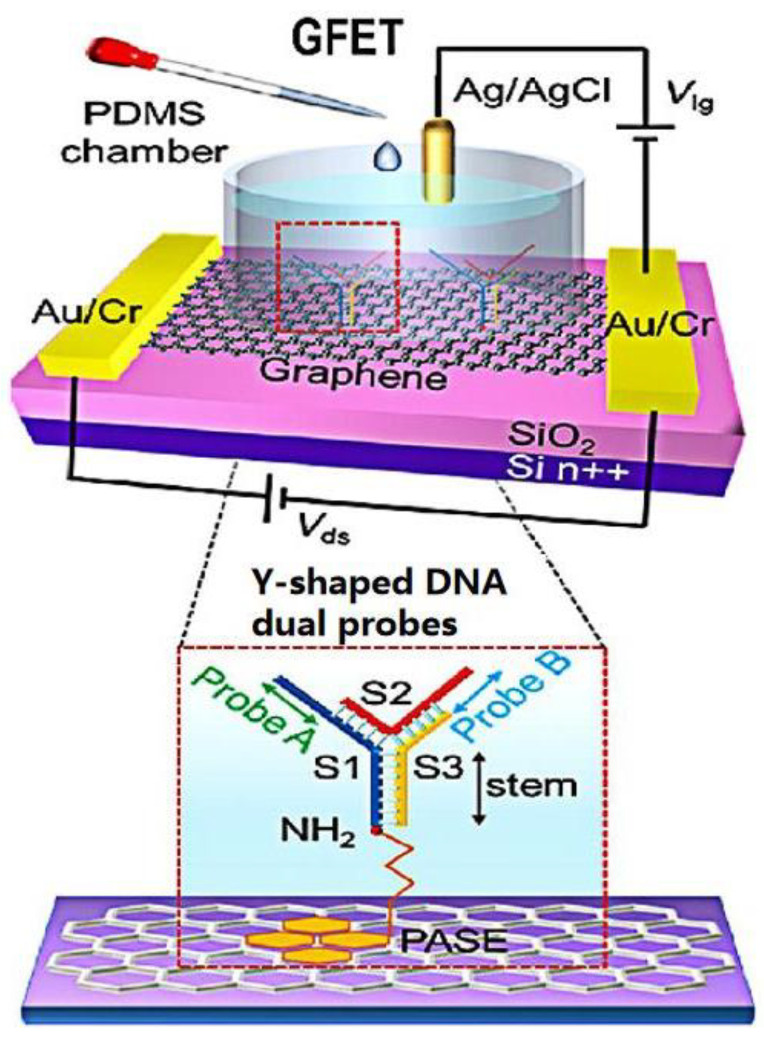
Schematic diagram of a Y-dual probe GFET biosensor. The dotted box is the structural schematic diagram of Y-shaped DNA dual probes. Reprinted from [54], with permission from the American Chemical Society.

In recent years, the development of structural DNA nanotechnology has provided an accurate and controllable method for synthesizing various DNA nanostructures with specific functions. Different DNA nanostructures have different properties for biosensors. Compared with the single-probe nucleic acid hybridization detection method, the two recognition sites of dual probes can improve the sensitivity of virus detection. Wei’s group [54] developed a direct acid nucleic assay using a GFET with Y-shaped DNA dual probes (Figure 6). These Y-shaped DNA dual probe GFET biosensor could simultaneously identify ORF1ab and N gene regions, improving the sensitivity for identifying SARS-CoV-2. The synergistic effect of the two recognition sites of Y-shaped DNA dual probes improved the combination of DNA dual probes and targets. Therefore, the Y-type dual-probe GFET biosensor with an average of 40 s for response speed had excellent performance in terms of diagnosis time and detection limit.

**Figure 7:**
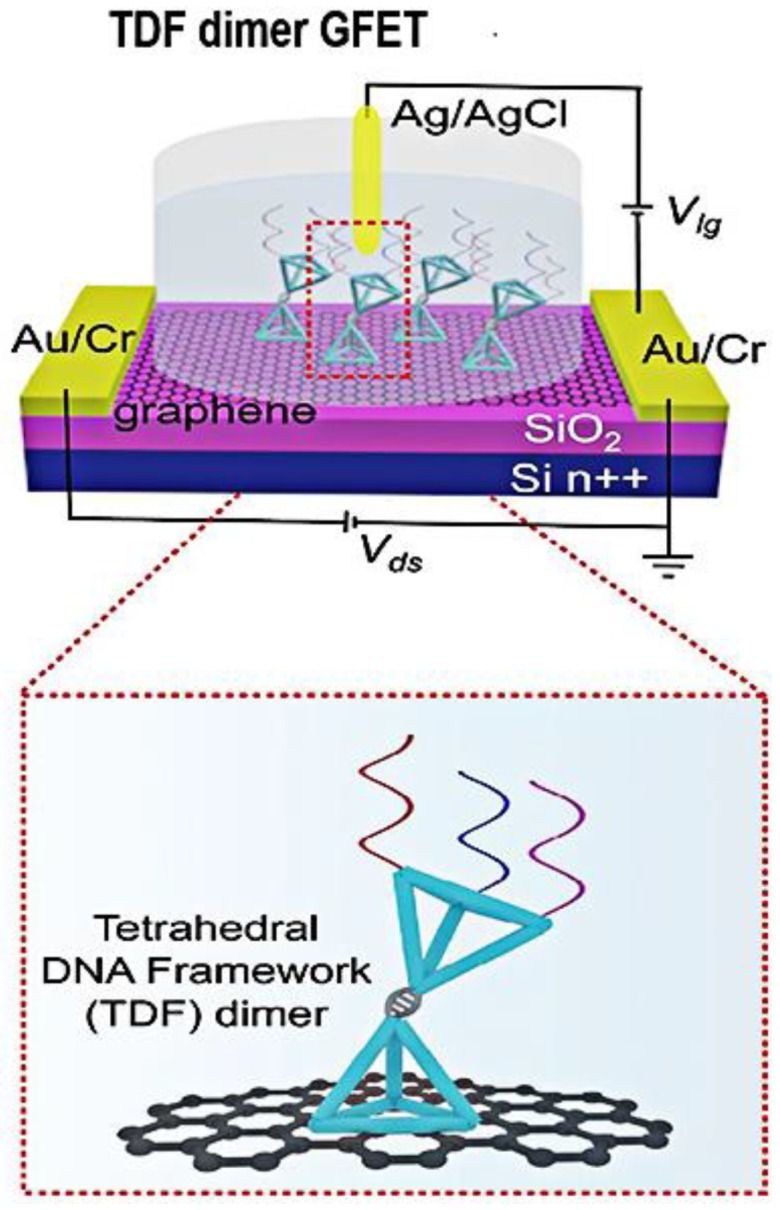
Schematic diagram of the triple-probe TDF dimer GFET sensor for SARS-CoV-2 RNA testing. The dotted box is the structural schematic diagram of TDF dimer. Reprinted from [55], with permission from the American Chemical Society.

**Figure 8:**
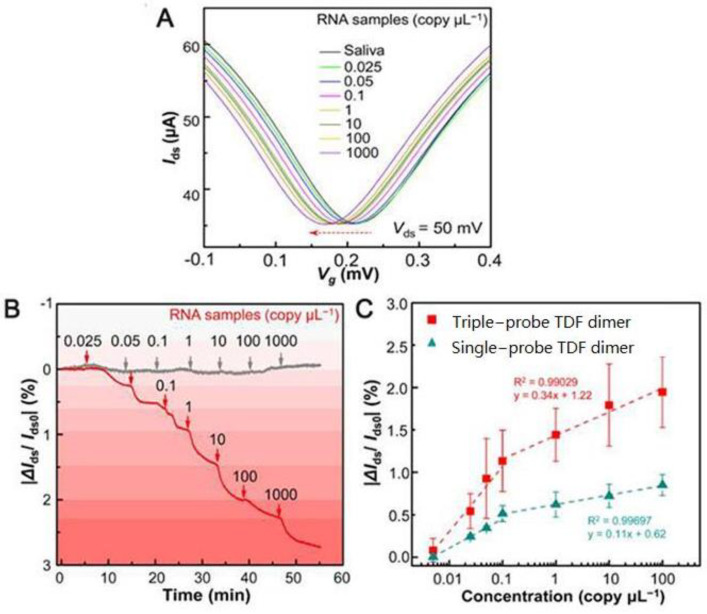
SARS-CoV-2 RNA testing. (**A**) Transfer curve measurement of adding different concentrations of target RNA (*I*_ds_−*V*_g_ response curve). (**B**) Real-time |Δ*I*_ds_/*I*_ds0_| response upon different concentrations of target RNA (red line, modified with triple-probe TDF dimer; gray line, without immobilized probes). (**C**) |Δ*I*_ds_/*I*_ds0_| responses of single- and triple-probe TDF dimer GFET sensors to different concentrations of target RNA. Reprinted from [55], with permission from the American Chemical Society.

Graphene surface-modified probe recognition sites greatly impact the performance of GFET biosensors. Therefore, improving the structural design of the probe is an important factor. Creatures with a multi-tentacle structure have strong olfactory sensitivity and capture and hunting ability. Inspired by these organisms, the sensitivity of multi-probe sensors can often be improved. Wei’s team designed the DNA nanostructure as a probe-tunable TDF dimer. The synergistic action of three probes improves the binding affinity and the sensitivity of the GFET biosensor. Wei’s group modified the GFET biosensor with a triple-probe tetrahedral DNA framework (TDF) to study its detection performance for SARS-CoV-2 RNA [55]. The triple-probe TDF dimer was modified on the surface of the graphene channel to form reaction targets (Figure 7). The sensor had highly specific recognition of RNA in the SARS-CoV-2 ORF1ab gene, RdRp gene, and E gene regions. This study found that the synergistic effect of triple probes improved the binding affinity and sensitivity of the sensor. As shown in Figure 8, under the same conditions, the response of the triple-probe TDF dimer was faster than that of dual-probe and single-probe TDF dimer sensors. The sensor identified all 14 positive cases in 30 nasopharyngeal swabs within an average diagnosis time of 74 s, showing promising prospects for real-time and centralized detection screening.

### 3.3. Double Function of GFET Biosensors in Response to the Detection of SARS-CoV-2

Outstanding achievements have been made in detecting SARS-CoV-2 by single-response nucleic acid hybridization and antigen–antibody-specific reactions. The dual response of GFET biosensors to detecting SARS-CoV-2 has also received attention. Ke ’s group [56] reported a highly sensitive, specific, and convenient bi-functional GFET biosensor for detecting SARS-CoV-2 with detection limits as low as ~0.1 and ~1 fg·mL^−1^. The research group immobilized the SS-DNA probe or SARS-CoV-2 antigen protein on the surface of the graphene channel through *π-π* interaction. Detection results could be obtained in 5–10 min using SS-DNA probe-specific hybridization with a viral RNA polymerase target or SARS-CoV-2 antigen–antibody-specific recognition to convert biochemical effects into electrical signals. In order to verify the sensitivity and accuracy of the sensor for COVID-19 diagnosis, 18 volunteers were recruited for nucleic acid detection and 9 were recruited volunteers for immune detection. The results are shown in Table 2. The results were consistent with the results for PCR detection, and the method was feasible.

**Figure 9:**
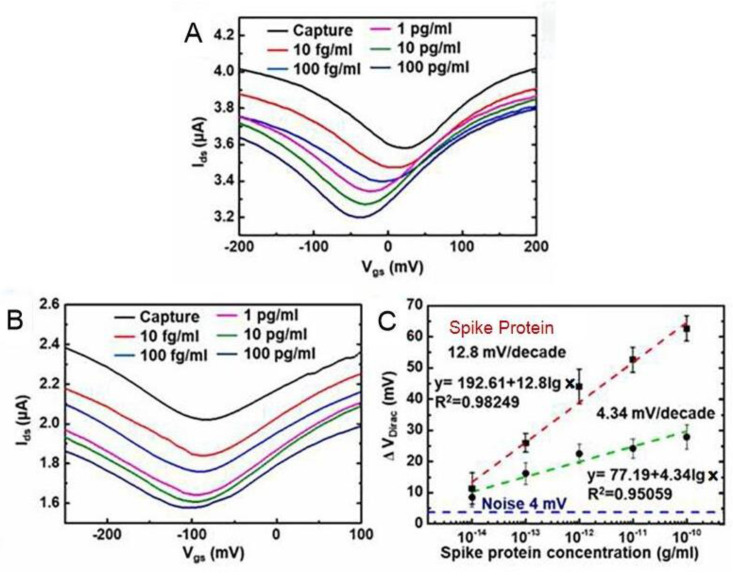
The SARS-CoV-2 spike protein concentrations dependent transfer curves of (**A**) GO/Gr FET biosensor and (**B**) Gr FET biosensor. (**C**) The SARS-CoV-2 spike protein concentrations dependent ΔVDirac shifts for both GO/Gr FET (red line) and Gr FET (green line) biosensors. Reprinted from [37], with permission from Elsevier.

Hwang’s group [57] was able to achieve high sensitivity by optimizing the crumpling ratio of the graphene sensing film. The results show that the crumpled GFET biosensor designed by Hwang’s group obtained good sensitivity and high reproducibility at a crumpling rate of about 55% [58]. The SARS-CoV-2 spike protein antibody and nucleocapsid protein antibody were immobilized on the surface of the graphene channel by *π-π* stacking, which could diagnose these two SARS-CoV-2 proteins at a lower detection limit. A field-effect transistor based on graphene oxide/graphene van der Waals heterostructures (GO/Gr heterostructure FET) was developed by Gao’s group [37]. Graphene oxide had abundant functional groups on its surface, and graphene oxide was superimposed onto graphene by *π-π* stacking, which enhances SARS-CoV-2 spike and nucleoprotein adsorption, improving the detection sensitivity of the sensor. The GO/Gr heterostructure FET sensor detects the SARS-CoV-2 protein in the range of 10 to 100 pg/mL with a limit detection as low as ~8 fg/mL. Meanwhile, as shown in Figure 9, the experimental data show ~3 × sensitivity enhancement compared with the GFET biosensor, which indicates its great potential for practical references in diagnosing SARS-CoV-2.

## 4. Other Types of Biosensors to Detect SARS-CoV-2

**Figure 10:**
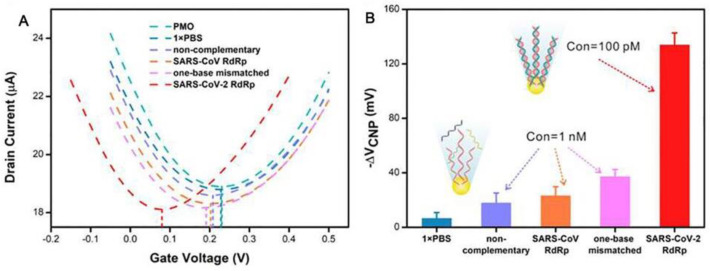
Excellent analytical performance of the COVID-19 GFET nanosensor. (**A**) Transfer curves upon incubation with PBS and nonspecific sequences including 1 nM non-complementary, SARS-CoV RdRp, and one-base mismatched RNA. (**B**) Variation of VCNP at detection of blank and three nonspecific sequences. Reprinted from [60], with permission from Elsevier.

As the COVID-19 outbreak continues, testing methods are critical to control the spread of the disease, and many types of biosensors have played an important role in detecting COVID-19. Biosensors are ideal for providing alternative and reliable clinical diagnosis solutions, real-time detection, and continuous monitoring. The presence of biosensors improves the efficiency of detection. For example, Qiu’s group [3] reported a dual-functional plasmonic biosensor that combines the plasmonic photothermal (PPT) effect and localized surface plasmon resonance (LSPR) sensing transduction. The localized PPT heat can elevate the in situ hybridization temperature, exhibiting a high sensitivity toward SARS-CoV-2 sequences with a lower detection limit to the concentration of 0.22 pM. Fabiani’s group [59] combined carbon black nanomaterial-modified screen-printed electrodes with magnetic beads (mb), developing an electrochemical immunoassay-based method to detect SARS-CoV-2. Rapid and accurate detection of the SARS-CoV-2 protein in saliva was established. Li’s group [60] designed a gold nanoparticle (AuNP)-decorated GFET nanosensor. The nanosensor could obtain detection results of COVID-19 patients within 2 min. As shown in Figure 10, the sensor was found to have high specificity for SARS-CoV-2 RNA detection and could accurately distinguish between SARS-CoV and SARS-CoV-2.

**Figure 11:**
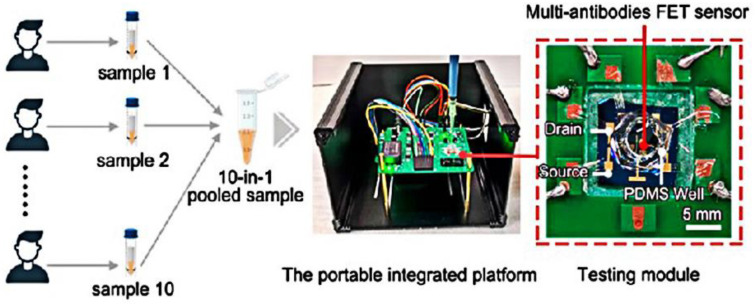
The portable integrated platform developed by the research and development of 10-in-1 COVID-19 antigen detection, processing diagrams and photos. The red dashed box indicates one packaged multiantibody FET sensor using a printed circuit board substrate. A polydimethylsiloxane well was stamped above the graphene channel to hold the analyte solution. Reprinted from [62], with permission from the American Chemical Society.

Wei’s group [61] developed an electro-enrichable liquid gate FET functionalized with tetrahedral DNA nanostructures (TDNs) for direct detection of the SARS-CoV-2 nucleic acid. In November of the same year, the same group developed high-precision 10-in-1 multiantibody FET sensor pool testing, which could detect different configurations of the SARS-CoV-2 spike S1 protein [62]. The multiantibody FET sensor was able to capture three different spatial structures, which greatly improved the recognition efficiency for the spike protein as well as the sensitivity of the sensor. Due to its highly accurate characteristics, this group developed a portable integrated platform, realizing 10-in-1 antigen pool detection, reducing detection costs, and improving testing capabilities (Figure 11).

**Figure 12:**
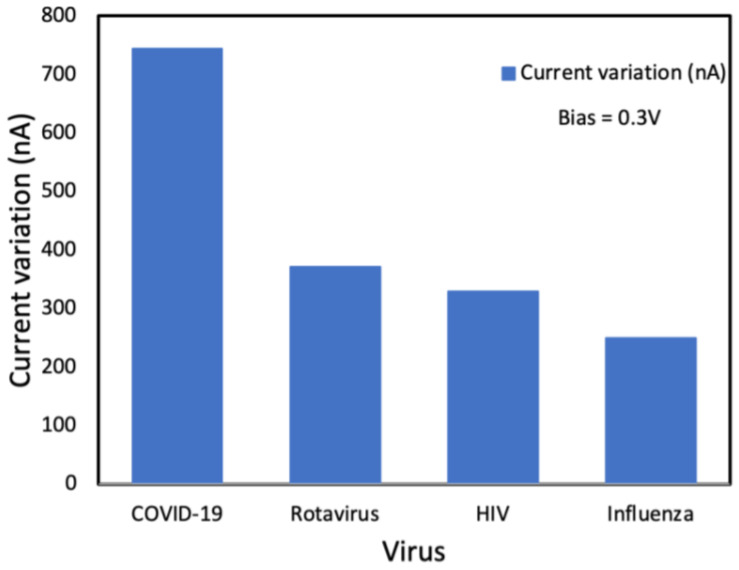
Change in the electrical drain current for different types of viruses.

A silicon nanowire field-effect transistor (SiNW-FET) biosensor functionalized with the SARS-CoV-2 spike protein antibody was developed by Wasfi’s group [63]. The selection of a SiNW-FET for COVID-19, influenza, rotavirus, and HIV was analyzed. As shown in Figure 12, the electrical signal changed significantly when the sensor was exposed to SARS-CoV-2, indicating that the SiNW-FET biosensor is highly selective to SARS-CoV-2 and has the potential to diagnose COVID-19.

## 5. Conclusions

This review mainly describes three types of GFET biosensors for detecting SARS-CoV-2. The GFET biosensors can quickly identify SARS-CoV-2 within ultra-low detection limits by specifically recognizing the SARS-CoV-2 protein antigen, antibody, or nucleic acid. Graphene is used as a sensor channel to improve the surface area and biocompatibility of sensor components. These functionalized GFET biosensors will selectively bind to SARS-CoV-2, showing excellent sensitivity and specificity. In addition, the modification of nanoparticles and the design of double probes and triple probes can significantly improve the performance of the sensor.

GFET biosensors have potential in the high-sensitivity detection of various analytes, pH values, various bacteria and viruses, chemicals, and pollutants. Therefore, GFET biosensors are expected to become an ideal multi-selective, multifunctional biological detection platform. GFET biosensors have the advantages of fast response and strong integration ability, so they are expected to be combined with readable signal equipment to be used in hospitals, clinics, and even at home or in other high-traffic areas. However, the formation of high-quality graphene is very complex and also very expensive. GFET biosensors are also susceptible to water molecules present in the atmosphere. It is difficult to detect analyte binding beyond the Debye length in the physiological environment. The Debye length problem remains an entrenched obstacle. But we believe that more research will be conducted in this field for future medical monitoring technology.

## Figures and Tables

**Table 1:** Methods for SARS-CoV-2 detection/diagnosis.

Methods	Target	Sample or Medium	Lod	Response Time	Ref.
Paper-based electrochemical biosensor	SARS-CoV-2 antibody	Serum	~6.4 × 10^−12^ M	30 min	[12]
Current–voltage electrochemical assay	SARS-CoV-2 RNA	Nasopharyngeal swabs	6.9 copy/μL	5 min	[47]
Chemiluminescent Immunoassay	SARS-CoV-2 antibody	Reaction mixture	4.6 μM	48 min	[48]
qRT-PCR	SARS-CoV-2 RNA	Nasopharyngeal swabs	11.2–21.3 copy/reaction	~60 min	[49]
BN-GO gel FET biosensor	SARS-CoV-2 N-protein	Buffer	0.00001 pg/mL	<4 min	[13]
GFET biosensor	SARS-CoV-2 antigen	Buffer	0.001 pg/mL	<1 min	[50]

**Table 2:** Nucleic acid analysis of COVID-19 patients and healthy subjects ^a^.

	**Patient 1**	**Patient 2**	**Patient 3**	**Patient 4**	**Patient 5**	**Patient 6**	**Patient 7**	**Patient 8**	**Patient 9**
Δ*R*/*R*_0_ (%)	−9	−5.8	−2.8	−3.6	−5.9	−8.9	−6.2	−3.1	6.1
GFET results	+	+	+	+	+	+	+	+	+
Agreement	Yes	Yes	Yes	Yes	Yes	Yes	Yes	Yes	Yes
	**Patient 10**	**Health 1**	**Health 2**	**Health 3**	**Health 4**	**Health 5**	**Health 6**	**Health 7**	**Health 8**
Δ*R*/*R*_0_ (%)	−5.4	1.6	−0.3	2	3.2	4.1	5.3	2.9	2.2
GFET results	+	−	−	−	−	−	−	−	−
Agreement	Yes	Yes	Yes	Yes	Yes	Yes	Yes	Yes	Yes

^a^ The “Cutoff value” was set at −1; “+” represents positive, and “−” represents negative; “Yes” indicates that the GFET result is consistent with the clinical standard samples. Reprinted from [56], with permission from Springer Nature.

## Data Availability

Not applicable.
